# Manipulating the contribution of approach-avoidance to the perturbation of economic choice by valence

**DOI:** 10.3389/fnins.2013.00228

**Published:** 2013-12-04

**Authors:** Nicholas D. Wright, Laurel S. Morris, Marc Guitart-Masip, Raymond J. Dolan

**Affiliations:** Wellcome Trust Centre for Neuroimaging, Institute of Neurology, University CollegeLondon, UK

**Keywords:** risk, loss, go-nogo, approach-avoidance, Pavlovian

## Abstract

Economic choices are strongly influenced by whether potential outcomes entail gains or losses. We examined this influence of outcome valence in an economic risk task. We employed three experiments based on our task, each of which provided novel findings, and which together better characterize and explain how outcome valence influences risky choice. First, we found that valence perturbed an individual's choices around that individual's base-level of risk-taking, a base-level consistent across time, and context. Second, this perturbation by valence was highly context dependent, emerging when valence was introduced as a dimension within a decision-making setting, and being reversed by a change in task format (causing more gambling for gains than losses and the reverse). Third, we show this perturbation by valence is explicable by low-level approach-avoidance processes, an hypothesis not previously tested by a causal manipulation. We revealed such an effect, where individuals were less disposed to choose a riskier option with losses when they had to approach (go) as opposed to avoid (nogo) that option. Our data show valence perturbs an individual's choices independently of the impact of risk, and causally implicate approach-avoidance processes as important in shaping economic choice.

## Introduction

The valence of potential outcomes, that is whether they reflect gains or losses, influences economic choices (Samuelson, 1963). A prevailing view is that outcome valence determines attitude to risk in a specific fashion, such that individuals are risk-averse with gains and risk-seeking with losses (Kahneman and Tversky, 1979). However, mounting evidence for an asymmetric processing of rewards and punishments in humans and other animals (Dayan and Seymour, 2008), has led to a questioning of this relationship. Instead, we suggest that valence influences choice independently of risk. This is evident in the fact that changing the task format can lead one group of subjects to gamble more for gains than losses, and another group to gamble more for losses than gains, where the overall amount of gambling was similar between the two groups (Wright et al., 2012). Here we sought to better characterize this behavior in two experiments. First, instead of comparing two different groups of subjects, we looked within individuals to characterize context dependence in responses to valence. Our rationale was that a priori it is unclear if the influence of risk and the influence of valence would be consistent within individuals, or if instead the pattern seen in our previous study only emerged at the group level. If the overall amount of risk-taking was consistent within individuals across contexts, this would provide a clearer basis for interpreting valence effects. Second, we compared two potential ways in which the influence of valence may emerge: either as a generic effect of trial type (e.g., trials with gains or trials with losses), or only when individuals were required to evaluate valence as an additional dimension in choice. Thus, in these two experiments we hypothesized that an individual would exhibit a consistent base-level of risk-taking, around which the introduction of valence as a dimension in choice would additionally perturb choice.

Furthermore, a biologically-based perspective on choice also suggests that one source for such a perturbation of choice by outcome valence derives, at least in part, from reflexive “Pavlovian” approach-avoidance processes. In simple instrumental tasks these underlie important valence effects, evident in a close coupling between punishment and nogo (avoid) responses, and between reward and go (approach) responses (Breland and Breland, 1961; Gray and McNaughton, 2000; Dickinson and Balleine, 2002; Guitart-Masip et al., 2011, 2012). However, in economic choice there exists only correlational evidence of a role for approach-avoidance, for example in longer reaction times for losses compared to gains (Dickhaut et al., 2003; Wright et al., 2012). Here we sought to directly manipulate approach-avoidance processes in economic choices, by harnessing the specific couplings between go/nogo and reward/punishment. We hypothesized that the perturbation induced by valence would depend on whether choice implies approach or avoidance.

We tested these hypotheses in three independent experiments (total *n* = 95), each using variations on an economic task that orthogonally manipulates valence and risk (measured as variance) in potential outcomes. We asked if individuals show a consistent base-level of risk-taking around which introducing valence as a dimension in choice perturbs choice (Experiments 1 and 2), and sought direct evidence implicating approach-avoidance in the perturbation of economic choice by valence (Experiment 3).

## Experiment 1: valence perturbs an individual's choice about their consistent base-level of risk-taking

In Experiment 1 we took a within individual approach to characterize context dependence in responses to valence, enabling new questions not addressable in between subject designs (Wright et al., 2012). Our rationale was that *a priori* it is unclear if the influence of risk and the influence of valence would be consistent within individuals, or instead in our previous study the observed pattern only emerged at the group level. If the overall amount of risk-taking was consistent within individuals across contexts, this would provide a clearer basis for interpreting valence effects. This within-individual approach enabled us to ask if there was a consistent base-level of risk-taking for an individual, despite changing task format so valence caused more gambling for gains than losses or the opposite; and whether valence perturbed an individual's choices around that base-level of risk-taking. Further, we could ask if the degree to which valence perturbed an individual's choices differed between contexts (i.e., it did not only reverse within individuals), which may suggest distinct aspects to the valence effect between these contexts.

### Participants

Thirty healthy participants took part (mean 24 years, range 17–38; 13 male; 29 right handed). All provided informed consent. University College London Ethic Committee approved the study.

### Task

Participants attended two separate sessions 5 days ± 0.2 (*SD*) apart. On each day they completed one of two versions of a risk task: on one attendance they completed an “accept/reject” task; and on the other a “selection” task. Work in separate groups of subjects suggested the accept/reject task led to more gambling with gains than losses, and the selection task to the reverse (Wright et al., 2012). Task order was counterbalanced between subjects (13 completed the accept/reject task first).

**Figure 1:**
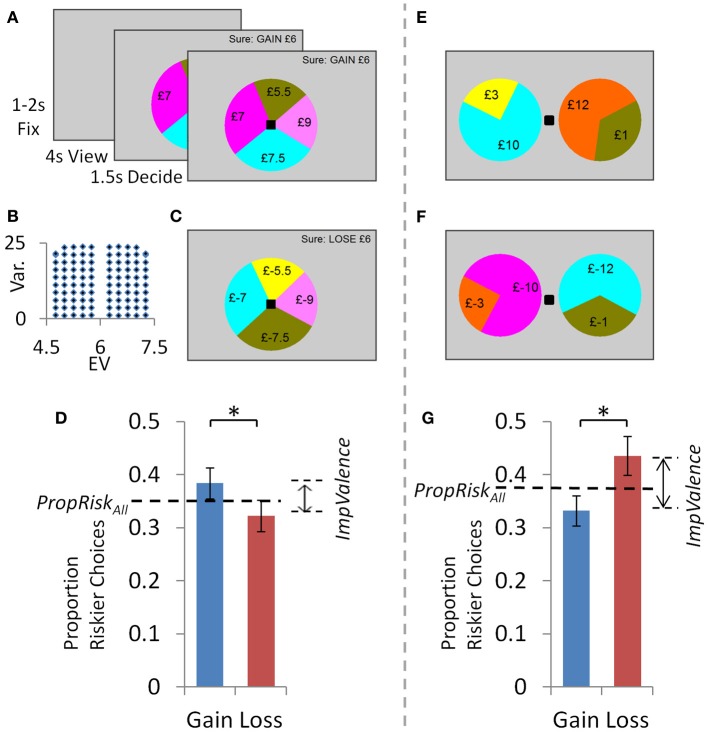
**Experiment 1: dissociating valence and risk related influences using task design.** In the “accept/reject” task: **(A)** In each “gain trial” individuals either accepted a lottery (four possible outcomes, all ≥0) or rejected and so received £6 for certain. **(B)** A set of 100 “gain trials” parametrically and orthogonally manipulated lottery risk and EV. Half the lotteries had an EV above the sure amount and half below, so the proportion of riskier choices indexed risk preference (PropRisk; risk-averse <0.5; risk-neutral = 0.5; risk-seeking >0.5). **(C)** Multiplying all “gain trial” amounts by −1 gave 100 “loss trials”. All 200 trials were presented in random order. **(D)** Behavior: Individuals were risk-averse overall (i.e., PropRiskall <0.5). Valence influenced choice, with more gambling for gains than losses. In the “selection” task there were: **(E)** 100 “gain trials” with parametric and orthogonal manipulation of difference in risk and EV between the two options; and **(F)** 100 “loss trials” created as before. However, here in each trial individuals were presented with two lotteries to consider and select between. **(G)** Behavior: risk-aversion overall was unaltered compared to the “accept/reject” task, but the direction of the valence effect was reversed. Error bars show s.e.m. ^*^P < 0.05.

In the “accept/reject” task (Figures 1A–C), in each trial participants chose to accept or reject a lottery (four possible outcomes) compared to a sure option (£6 in “gain trials”; £-6 in “loss trials”). Each trial began with a fixation cross presented for 1–2 s (mean 1.5 s); followed by viewing the options for 4020 ms; and finally a black square appeared to indicate participants had 1500 ms to input their choice by button press (press “G” for gamble or “S” for sure; the black square turned white when they chose). If participants did not respond they received £0 on a “gain trial” and the maximum loss possible on a “loss trial” (£-12). There were 100 “gain trials” [all possible outcomes between £0 and 12, details in Wright et al. (2012)] in which we parametrically and orthogonally manipulated the difference in risk (10 levels of difference in variance) and expected value (EV; 10 levels) between the two options in each trial (Figure 1C). This trial set was multiplied by −1 to give 100 “loss trials” (outcomes ≤ £0) that were perfectly matched in their parametric modulations of risk and EV. All 200 trials were presented in random order.

The “selection” task (Figures 1E,F) was identical except that on every trial individuals evaluated two simultaneously presented lotteries (each with two possible outcomes, all £0–12) and selected between them (with a left or right button press to select left or right lottery). Again in 100 “gain trials” we parametrically and orthogonally manipulated the difference in risk (10 levels) and EV (10 levels) between the two lotteries. As before, we multiplied all amounts by −1 to give 100 “loss trials”. All 200 trials were presented in random order.

Participants began each session with an endowment of £12. After the session, one “gain trial” and one “loss trial” were picked at random and their outcomes were added to this endowment to determine payment for that session. Participants could receive between £0 and 24 per session (i.e., £0–48 in total). Participants received all feedback and payment after the second attendance.

### Results

**Figure 2:**
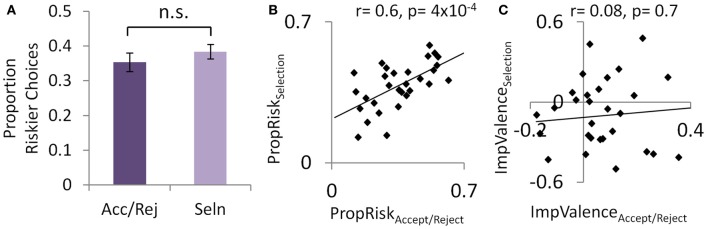
**Experiment 1: consistent base-level of risk-taking within individuals. (A)** Across subjects risk-taking was consistent between the accept/reject task and selection tasks, despite reversing the direction of the valence effect. **(B)** This consistent base-level of risk-taking was also seen within individuals. **(C)** The valence effect was not correlated within individuals between the two tasks.

Individuals' base-level of risk-taking was highly consistent across tasks, both within individuals (Figure 2B) and across subjects (Figures 1D,G and 2A). In both tasks, a simple metric for the influence of risk on choice is the proportion of riskier choices made (*PropRisk*; where risk-neutral = 0.5; risk-averse <0.5; risk-seeking >0.5). An individual's base-level of risk-taking was highly consistent between tasks (*r* = 0.6, *p* < 0.001; Figure 2B). This consistency was also shown across subjects, with risk aversion in both the accept/reject task [*PropRisk*_all_ 0.35 ± 0.15 (*SD*); one sample *t*-test against risk neutral, *t*_(29)_ = −5.47, *p* < 0.001] and the selection task [*PropRisk*_all_ 0.38 ± 0.11; one sample *t*-test against risk neutral, *t*_(29)_ = 5.56, *p* < 0.001], and this did not differ between tasks (paired samples *t*-test, *t* = −1.4, *p* = 0.17; Figure 2A).

Valence perturbed choice about this base-level of risk-taking in both tasks, but did so in opposite directions depending on task (Figures 1D,G). In the accept/reject task individuals gambled more for gains (*PropRisk*_gain_ 0.38 ± 0.16) than losses [*PropRisk*_loss_ 0.32 ± 0.16; mixed analysis of variance [ANOVA] with factors of 2 valence [gains, losses; within-subjects] by 2 task order [between subjects], main effect of valence *F*_(1, 28)_ = 6.9, *p* = 0.014; no interaction]. This effect was reversed in the selection task where individuals gambled more for losses (*PropRisk*_loss_ 0.44 ± 0.2) than gains [*PropRisk*_gain_ 0.33 ± 0.16; 2 valence by 2 order mixed ANOVA, main effect of valence, *F*_(1, 28)_ = 4.57, *p* = 0.041; no interaction]. However, the degree to which valence influenced an individual's choices (*ImpValence = PropRisk*_gain_*-PropRisk*_loss_) was not consistent within individuals between the tasks (*r* = 0.08, *p* = 0.66; Figure 2C; i.e., not negatively correlated), suggesting the possibility of distinct aspects to the valence effect between these contexts.

A further dissociation of risk and valence effects was suggested between subjects by the lack of correlation between measures relating to each on individuals' choices (*PropRisk*_all_ v. *ImpValence*: accept/reject, *r* = 0.00; *p* = 0.99; selection, *r* = −0.25; *p* = 0.18; Figure S1). In both tasks reaction times were slower to choose losses than gains, and behavioral modeling of choice confirmed that EV, risk, and valence influenced choice (Supplementary Results).

## Experiment 2: introducing valence as a dimension in choice

We next tested individuals in a within-subjects design on the accept/reject task across three sessions: one in which all trials involved only gains (*GainAlone*), one with losses alone (*LossAlone*), and one where gain and loss trials were randomly interleaved as before (*CombinedValence*). This enabled us to compare two potential ways in which a perturbation by valence may emerge. One was as a generic effect of trial type, such that the valence effect would be seen between sessions (e.g., more gambling in the *GainAlone* than *LossAlone* session) as well as within the *CombinedValence* context. Alternatively, risk-taking could be consistent between sessions (i.e., equal in *GainAlone* and *LossAlone*), and instead the valence perturbation could emerge in the *CombinedValence* context where interleaved gain and loss trials required individuals to evaluate valence as an additional dimension in choice.

### Participants

Data from 26 participants (mean 25 years, range 19–32; 10 male; 25 right handed) were included, with three further participants excluded (one deterministically only rejected and two made a high proportion of non-responses).

### Task

Participants attended on three separate occasions (each 7 ± 1 days apart). They completed a different version of the accept/reject task on each attendance: once in a *GainAlone* context with the set of 100 gains trials presented twice (i.e., 200 trials total); once in a *LossAlone* context with the set of 100 loss trials presented twice (200 trials total); and once in a *CombinedValence* context with 100 gain trials and 100 loss trials (i.e., as in Experiment 1; 200 trials total). Task order was counterbalanced between subjects (≥4 participants for each of six possible orders). In order to keep the range of possible outcomes between £0 and 24 in all three contexts, in the*CombinedValence* context there was an endowment of £12 to which one gain and one loss trial outcome was added as above; *GainAlone* context a £0 endowment to which two gain trial outcomes were added; and in the *LossAlone* context a £24 endowment to which two loss trial outcomes were added. Participants received all feedback and payment after the third attendance.

### Results

**Figure 3:**
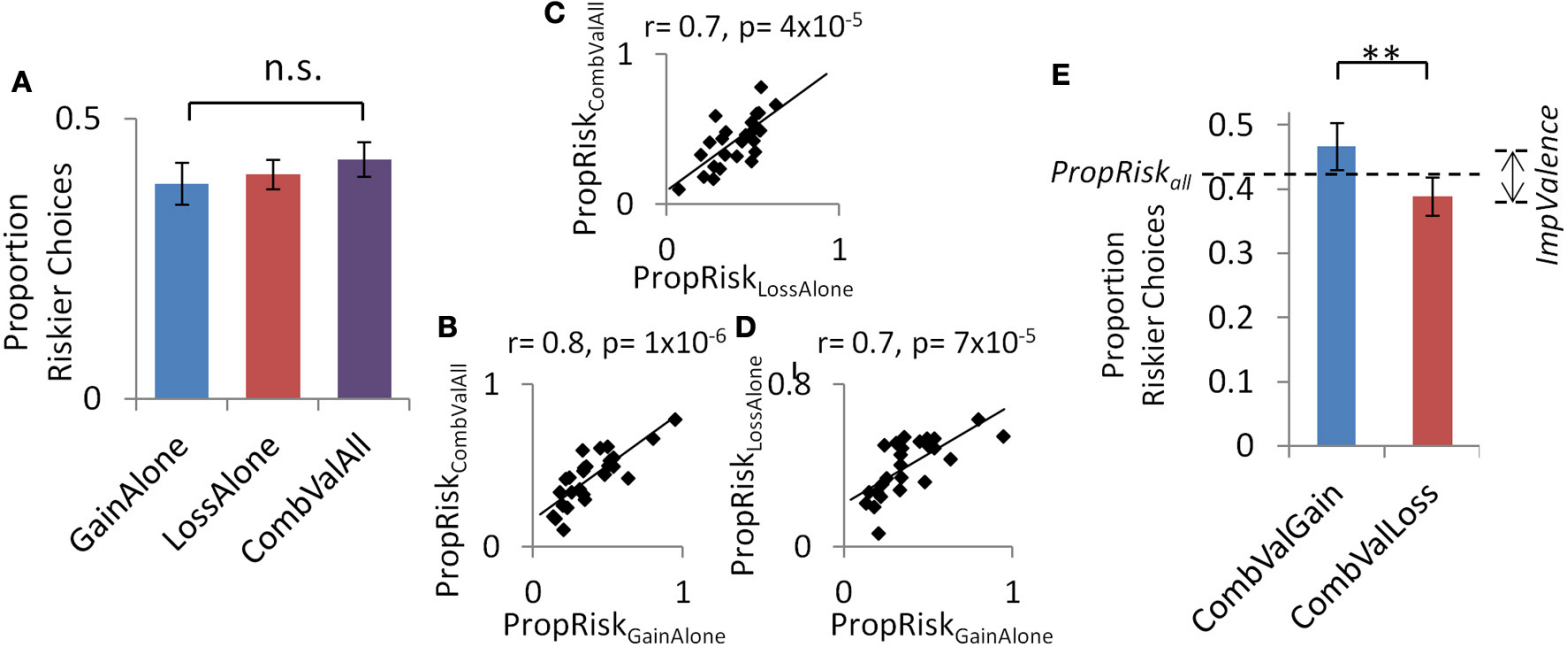
**Experiment 2: consistent base-line risk-taking within individuals and additional perturbation by introducing a valence as a dimension in choice.** A consistent base-level of risk-taking in the accept/reject task was seen in the three contexts **(A)** across subjects and **(B–D)** within individuals. **(E)** The perturbation by valence emerged within the setting with both gain and loss trials. Error bars show s.e.m. ^**^p <0.005.

Individuals' base-level of risk-taking was highly consistent across all three contexts. This was seen within individuals, with *PropRisk*_all_ highly correlated across the three contexts (all pairwise comparisons between contexts *r* > 0.7 and *p* < 0.001, further details in Figures 3B–D and Supplementary Results). Across subjects, there was no difference in the proportion of risky choices (*PropRisk*_all_) between the three contexts [*CombinedValence* 0.43 ± 0.2; *LossAlone* 0.4 ± 0.14; *GainAlone* 0.38 ± 0.2; One-Way ANOVA, *F*_(2, 50)_ = 1.56, *p* > 0.2; no significant pairwise *t*-test; Figure 3A] and there was risk-aversion in all three contexts (Supplementary Results).

However, introducing valence as a dimension in choices in the *CombinedValence* context (i.e., the standard accept/reject task in Experiment 1) revealed exactly the same perturbation by valence about the base-level of risk-taking seen before, such that individuals gambled more for gains than losses (*CombinedValence: gain* 0.47 ± 0.2; *loss* 0.39 ± 0.16); [*t*_(25)_ = 3.02, *p* = 0.006; Figure 3E]. For completeness, we note there was no difference in rates of gambling between the *CombinedValence* loss trials and *LossAlone* context [*t*_(25)_ = 0.7, *p* = 0.5], but there was a difference in gambling between the *CombinedValence* gain trials and *GainAlone* context [*t*_(25)_ = 3.2, *p* = 0.003].

Reaction times also suggested that rather than a generic effect of trial type, instead the valence perturbation emerged in the CombinedValence contex where individuals evaluated valence as an additional dimension in choice. RTs did not differ between the GainAlone (462 ± 88 ms) and LossAlone [480 ± 112 ms; *t*_(25)_ = −0.8, *p* = 0.4] sessions, but there was a large effect of valence in the CombinedValence context [gains 485 ± 100 ms; losses, 560 ± 109 ms; *t*_(25)_ = −6.3, *p* < 0.001]. Further, whilst there was no difference between the types of gain trial [*t*_(25)_ = 1.04, *p* > 0.3] there was between the types of loss trial [*t*_(25)_ = 3.25, *p* = 0.003]. However, we note that when analysing these RT data in an ANOVA with factors of 2 context type (combined and separate) by 2 valence (gain and loss), although there were main effects of valence [*F*_(1, 25)_ = 17.09, *p* < 0.001] and context type [*F*_(1, 25)_ = 7.96, *p* = 0.009], the interaction showed only a trend-level significe [*F*_(1, 25)_ = 3.74, *p* = 0.06]. Further details in Supplementary Results.

## Experiment 3 manipulating approach-avoidance processes in the perturbation by valence

**Figure 4:**
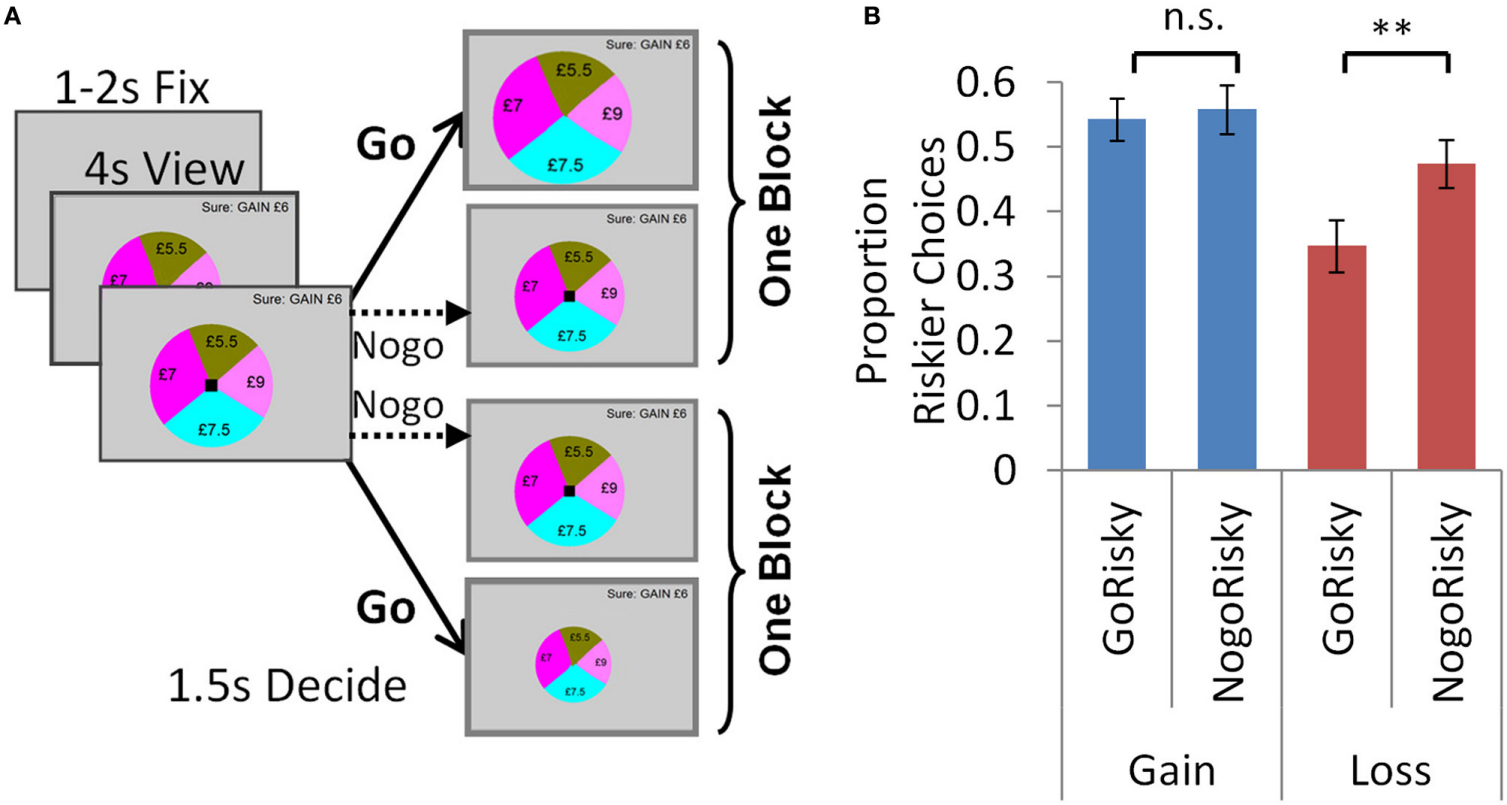
**Experiment 3a: go-nogo manipulation in the accept/reject task. (A)** Trials were identical to the accept/reject task above, except here participants could either go (button press) or nogo (no button press). In one block (GoRiskier), participants had to go to accept the risky option or nogo to reject. In the other block (NogoRiskier), participants had to nogo to accept the risky option or go to reject it. **(B)** Individuals were averse to accepting (approaching) the risky option with losses, and chose it less frequently when they had to approach it (go) than avoid it (nogo). There was no effect of action in the gain trials. Error bars show s.e.m. ^**^p < 0.005.

**Figure 5:**
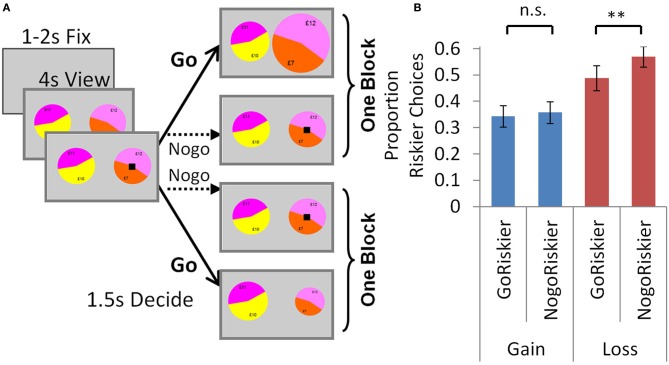
**Experiment 3b: go-nogo manipulation in the selection task. (A)** To indicate in each trial which lottery would be selected by a go and which by a nogo response, the black square signaling the choice period appeared on one of the lotteries, and individuals responded by either accepting or rejecting it. In one block, individuals were instructed to go to accept the lottery with the black square. In the other block, individuals would no-go to accept the lottery with the black square. In each trial the lottery to be accepted or rejected could be either the riskier or less risky lottery, so choices may again be parsed as either: go to accept the riskier option (GoRiskier); or nogo to accept the riskier option (NogoRiskier). **(B)** For the go-nogo manipulation we see the same pattern as in the previous experiment: individuals chose the risky option with losses less frequently when they had to go to accept (GoRiskier) than nogo to accept (NogoRiskier). There was no effect of action in the gain trials. Error bars show s.e.m. ^**^p < 0.005.

In Experiment 3 we asked if the perturbation by valence, and its context-dependence shown in Experiment 1, were derived from approach-avoidance processes. Here we sought to directly manipulate approach-avoidance processes by harnessing the specific “Pavlovian” couplings between go/nogo and reward/punishment. We examined this go/nogo manipulation in separate groups who undertook modified versions of the accept/reject task (Experiment 3a) and selection task (Experiment 3b), which can be readily compared in Figures 4, 5.

### Experiment 3a: go-nogo accept/reject task

#### Participants

Data from 17 participants (mean 23 years, range 19–35; 6 male; 15 right handed) were included (two further deterministic participants who always gambled in gains and never in losses were excluded).

#### Task

Each trial was identical to the accept/reject task above except for the action by which individuals expressed their choice. As before, they viewed the options for 4020 ms, after which the black square appeared to signal they had 1500 ms to choose. Instead of indicating choice by two different button presses, here participants could either respond with go (button press) or nogo (no button press) (Figure 4A). Participants undertook two separate blocks. In one block (*GoRiskier*), participants had to go to accept the risky option (upon which it increased in size) or nogo to reject. In the other block (*NogoRiskier*), participants had to nogo to accept the risky option or go to reject it (upon which it decreased in size). The order of blocks was counterbalanced between subjects (9 participants GoRiskier block first).

This experiment used a set of 49 “gain trials” (as above but with 7 levels of EV and 7 levels of variance). Forty nine loss trials were created by multiplying all amounts by −1. In each block the 49 gain and 49 loss trials were randomly interleaved as above (i.e., 98 trials per block, giving 196 in the whole session). Payment was as above (endowment of £12, plus the outcomes of one gain and one loss trial; range of possible outcomes £0–24), with all feedback at the end of the session.

#### Results

Individuals chose the risky option less for losses than gains overall (Figure 4B; ANOVA below), which we hypothesized to arise because individuals were averse to accepting the risky option with losses. We predicted that this effect would be exacerbated by the go-nogo manipulation, and therefore individuals would choose the risky option with losses less frequently when they had to approach (go) rather than avoid it (nogo). We observed this precise effect: individuals chose the risky option with losses less frequently when they had to go to accept (in the GoRiskier block *PropRisk*_loss_ 0.35 ± 0.16) than nogo to accept [in the NogoRiskier block *PropRisk*_loss_ 0.47 ± 0.16; *t*_(16)_ = −3.39, *p* = 0.004; Figure 4B]. This effect of action was selective, with no difference seen in the gain trials [GoRiskier block *PropRisk*_gain_ 0.54 ± 0.14; NogoRiskier block *PropRisk*_gain_ 0.56 ± 0.17; *t*_(16)_ = −0.44, *p* = 0.67]. To summarize, a 2 valence (gain, loss) × 2 action (GoRiskier, NogoRiskier) ANOVA with PropRisk as dependent variable showed a main effect of action [*F*_(1, 16)_ = 5.12; *p* = 0.038]; main effect of valence [*F*_(1, 16)_ = 11; *p* = 0.004]; and an interaction [*F*_(1, 16)_ = 8.98; *p* = 0.009].

### Experiment 3bs: go-nogo selection task

#### Participants

Data from 22 participants (mean 24 years, range 18–39; 10 male; 18 right handed) were included (two further participants who did not understand the task were excluded).

#### Task

Each trial here was identical to the selection task above except for the action by which individuals expressed their choice. Here, for the go-nogo manipulation it was necessary to indicate in each trial which lottery would be selected by a go and which by a nogo response. To achieve this, the black square signaling the 1500 ms choice period appeared on one of the lotteries, and individuals responded by either accepting or rejecting it (Figure 5A). In one block, individuals were instructed to go to accept the lottery with the black square (upon which it increased in size) or nogo to reject and choose the other lottery. In the other block, individuals would either no-go to accept the lottery with the black square; or go to reject it (upon which it decreased in size).

The order of blocks was counterbalanced between subjects (10 participants' first block was to go to accept the lottery with the black square). We used a set of 32 gain trials (as above but with 4 levels of ΔEV and 8 levels of ΔVar), shown twice in each block (once with the black square on the left lottery, once on the right). Loss trials were created as above. Each block contained 64 gain and 64 loss trials randomly interleaved (i.e., 128 trials per block, giving 256 in the whole session). Payment and feedback was as in Experiment 3a.

#### Results

Here in each trial the lottery to be accepted or rejected could be either the riskier or less risky lottery, such that choices could again be parsed as either: go to accept the riskier option (GoRiskier) and nogo to reject; or nogo to accept the riskier option (NogoRiskier) and go to reject. For the go-nogo manipulation we replicate the pattern seen in Experiment 3a: individuals chose the risky option with losses less frequently when they had to go to accept (GoRiskier *PropRisk*_loss_ 0.49 ± 0.2) than nogo to accept [NogoRiskier *PropRisk*_loss_ 0.57 ± 0.2; *t*_(21)_ = 3.1, *p* = 0.005; Figure 5B]. Again, this effect of action was selective, with no difference seen in the gain trials [GoRiskier *PropRisk*_gain_ 0.34 ± 0.2; NogoRiskier *PropRisk*_gain_ 0.36 ± 0.2 (*SD*); *t*_(21) = −0.8_, *p* = 0.4]. To summarize, a 2 valence (gain, loss) × 2 action (GoRiskier, NogoRiskier) ANOVA with PropRisk as dependent variable showed a main effect of action [*F*_(1, 21)_ = 7.9, *p* = 0.01]; main effect of valence [*F*_(1, 21)_ = 6.1, *p* = 0.02] and an interaction [*F*_(1, 21)_ = 6, *p* = 0.02]. These results may be most clearly compared with those of Experiment 3a by comparing Figures 4, 5.

## Discussion

Our data reveal a striking consistency in individuals' base-level of risk-taking, across time and task context, and around which valence perturbed choice. This perturbation by valence was highly context dependent, being both reversible by changing task format (i.e., more gambling for gains than losses and the reverse), and also emerging only when valence was a choice dimension within a decision-making setting but not as an intrinsic valence-dependent risk preference. Furthermore, the degree to which valence perturbed an individual's choices differed between contexts, suggesting different aspects to the valence effect between contexts. Crucially, our data provide an explanation for this influence of valence on economic choices, founded in the likely biological bases of reward/punishment asymmetries. The causal data reveal a contingency between nogo and punishment that implicate “Pavlovian” approach-avoidance processes in the perturbation of economic choice by valence; and such approach-avoidance processes can also explain the context dependence in the effect of valence.

The consistent base-level of risk-taking shown here by individuals concurs with previous work using an assay of risk preference (Andersen et al., 2008), although has not to our knowledge been shown across such marked contextual manipulations (Experiments 1 and 2). With respect to characterizing the perturbation by valence, the reversibility we see here (i.e., more gambling for gains than losses and the reverse; Experiment 1) replicates such behavior in separate groups, and fits with an hypothesis that valence and risk in economic stimuli exert independent influences on choice (Wright et al., 2012). That a valence perturbation emerges when it features as a dimension within a decision-making setting (Experiment 2), suggests the possibility that juxtaposing trials of contrasting valence lends valence a greater salience as a dimension in choice. An alternative suggested by our data is that losses generate an avoidance response that can only be manifested when gains are an option in the decision-making environment.

Our data also link the influence of valence in economic choice to a broader biological literature on valence effects, and in particular to the neural basis of reward/punishment asymmetries (Experiment 3). In simple instrumental tasks, a role for reflexive approach-avoidance processes in the effect of valence on choice is well established, with evidence for a close coupling between reward and go (approach) responses, and between punishment and nogo (avoid) responses (Breland and Breland, 1961; Gray and McNaughton, 2000; Guitart-Masip et al., 2011, 2012). Here we harnessed the specific couplings between go/nogo and reward/punishment, showing that individuals chose a riskier option less often with losses when the instrumental requirement was to approach (go) as opposed to avoid (nogo). This is in line with previous correlational RT data, where RTs were longer for losses than gains in economic choices (Dickhaut et al., 2003; Wright et al., 2012). The observation that stimuli signaling loss induce avoidance can explain puzzling behavior across a variety of tasks. Previous work showed that framing a sure option as a loss can bias individuals to avoid a sure option and choose a gamble option instead (De Martino et al., 2006), a bias also elicited by incidentally presenting aversive conditioned stimuli with the sure option (Guitart-Masip et al., 2010). Avoidance of stimuli containing losses also explains a disposition not to choose mixed gambles that contain losses along with gains (Tom et al., 2007).

An approach-avoidance framework also helps explain the context dependence for losses seen between our “accept/reject” and “selection” tasks (Figure 1). Context determines animals' responses to aversive stimuli, such that, depending on what is called the defensive distance, rats in different contexts may respond to threat by fleeing, freezing or even fighting (Blanchard and Blanchard, 1988; Dayan and Seymour, 2008). Thus, we suggest that in both tasks loss induces avoidance and that context determines how exactly this is expressed. In the accept/reject task individuals decide to accept or reject a lottery, and when it contains losses they express avoidance by withdrawal. The effect of the go-nogo manipulation was entirely consistent with this in the accept/reject task (Figure 4), and also when individuals were required to accept or reject an option in the go-nogo manipulation in the selection task (Figure 5). However, such a withdrawal response does not explain the valence effect in the selection task, where individuals gamble more overall with losses than gains.

In the selection task we previously suggested that because individuals had to select between two lotteries, and so could not express avoidance by withdrawal as in the accept/reject task, instead they could potentially escape losses by selecting the riskier option (Wright et al., 2012). Such an additional aspect to the response to loss in the selection task (e.g., analogous to fight or to flight) relative to the accept/reject task (e.g., withdrawal), is suggested here by an observation that the impact of valence within individuals did not correlate between tasks in Experiment 1 (Figure 2C). This lack of correlation is in contrast both to a strong correlation in individuals' base-level risk-taking between tasks (Figure 2B); and also to a consistent impact of valence seen previously when the accept/reject task was undertaken on 2 days (Wright et al., 2012). We note such a framework can explain context effects between our “accept/reject” task where each trial presented a different lottery to accept or reject, relative to the problems in the classic paper establishing Prospect Theory (Kahneman and Tversky, 1979) where each problem presented two options for individuals to select between. It also explains context effects in the same direction shown for “mixed gambles” (Ert and Erev, 2008), and comparisons between sure options (Jones et al., 1998).

Finally, we note that the go-nogo manipulation here did not reveal a contingency between approach and appetitive stimulus aspects, for example as suggested previously in RT correlations (Wright et al., 2012). One possibility is that the threat of losses may exert a relatively stronger effect, regarding which as noted before an approach-avoidance account is entirely consistent with the idea that losses have greater weight (“loom larger”) than gains (Wright et al., 2012), and this might be usefully explored in further work.

Our findings provide a new perspective on how losses influence economic choice. Our data show that valence perturbs individuals' choice about a base-level of risk-taking and that responses to valence are highly context dependent. Further, our data explain these findings as the result of asymmetric neural processes related to rewards and punishments (Dayan and Seymour, 2008). These findings are not predictable under prevailing behavioral economic theories (Kahneman and Tversky, 1979), but are consistent with a biologically-based account of choice.

## Author contributions

Nicholas D. Wright designed the study and Laurel S. Morris collected the data. Nicholas D. Wright, Marc Guitart-Masip and Laurel S. Morris analyzed the data. All authors contributed to the manuscript.

### Conflict of interest statement

The authors declare that the research was conducted in the absence of any commercial or financial relationships that could be construed as a potential conflict of interest.

## Supplementary material

The Supplementary Materialfor this article can be found online at: http://www.frontiersin.org/journal/10.3389/fnins.2013.00228/abstract
