# Supplementary material for: Manipulating the contribution of approach-avoidance to the perturbation of economic choice by valence
Source: Front Neurosci. 2013 Dec 4;7:228. doi: 10.3389/fnins.2013.00228 (PMC3849845; doi:10.3389/fnins.2013.00228)
Supplement: Supplementary file 1 [file DataSheet1.DOCX]

# Supplementary Materials:

# Manipulating the contribution of approach-avoidance to the perturbation of economic choices by valence

## Experiment 1

### Methods

All statistical tests were two tailed.

Reaction Times (RTs) were normalised as in Wright et al. (2012), and again findings were unaltered for “raw” or normalised RTs.

#### Behavioural modelling

Choice was modelled identically to Wright et al. (2012), using maximum likelihood analysis for individuals and comparing models with the Bayesian Information Criterion (BIC). In each trial the utilities (*U*) of both options were computed using one of the utility functions below.

We compared three “summary statistic” models. First, a simple mean only (MO) model (*U = EV*). Second, adding an influence of risk in a Mean-Variance (MV) model (*U = EV + Var*ρ*), where *ρ* is a free parameter. Third, adding an influence of valence in the winning Mean-Variance-Valence (MVV) model from our previous datasets, with a *ρ_gain_* parameter in gain trials and a *ρ_loss_* parameter in loss trials. We implemented Expected Utility, Prospect Theory and Cumulative Prospect Theory models detailed previously (Wright et al., 2012).

### Results

#### Reaction times

RTs in both tasks replicated our previous findings (Wright et al., 2012). Valence influenced RTs, with individuals slower to approach losses than gains [Accept/Reject, RT_gain_, mean 511 ± s.d. 79, RT_loss_, 551 ± 95 ms; *t_(29)_* = -4.07, p < 0.001; Selection, RT_gain_, 482 ± 118, RT_loss_, 570 ± 136 ms; *t_(29)_* = -7.97, p < 0.001; Fig. S2].

Regarding risk, we looked between subjects. Individuals' risk preference with losses (PropRisk_loss_) strongly predicted the RT bias (RT_riskier_ – RT_surer_) with losses [Accept/Reject, r = -0.38, p = 0.039; Selection, r = -0.76, p < 0.001]; and risk preference with gains (PropRisk_gain_) predicted the RT bias with gains [Accept/Reject, r = -0.51, p = 0.004; Selection, r = -0.46, p = 0.01; Fig S2].

Finally, the proportion of missed trials was low (accept/reject, 3 ± 4%; selection, 4 ± 6%) and unaltered between tasks (*t*_(29)_ = -0.5, p = 0.6).

#### Modelling of choice

We replicate our previous findings. Variance and valence consistently influenced choice, as shown by comparing our “summary statistic” models. In the accept/reject task the mean-only model (MO BIC=7385) was improved by adding risk (MV 6160), and the winning model also added valence (MVV 6144). This MVV model also outperformed Expected Utility, Prospect Theory and Cumulative Prospect Theory models. The same was seen in the selection task (MO 7564; MV 7939; MVV 6280). In absolute terms we can see that participants took account of EV, with the MO model correctly predicting 65%±s.d.8% of choices in the accept/reject and 62%±7% in the selection tasks (random choice would be 50%), and that this was improved further by the MVV model that correctly predicted 75%±s.d.10% of choices in the accept/reject and 77%±7% in the selection tasks.

## Experiment 2

### Results

Choice in the *CombinedValence* context (i.e. the standard accept/reject task in Experiment 1) again replicated previous results. Across subjects there was more gambling for gains than losses (*PropRisk_gain_* 0.47 ± 0.2; *PropRisk_loss_*, 0.39 ± 0.16; *t_(25)_* = 3.02, p = 0.006); risk-aversion overall (see below); risk-aversion in losses (one sample *t* test against risk neutral, *t_(25)_* = -3.63, p = 0.001); and risk-neutrality in gains [*t_(25)_* = -0.89, p = 0.38). Between subjects *PropRisk_all_* and *ImpValence* were not correlated (r = 0.30; p = 0.14).

Across subjects there was risk-aversion in all three contexts: *GainAlone* context (*PropRisk_all_* one-sample *t* test against risk neutral, *t_(25)_* = -3.0, p = 0.006); *LossAlone* context [*t_(25)_* = -3.6, p = 0.001], and the *CombinedValence* context [*t_(25)_* = -2.3, p = 0.032].

Between subjects, we note that risk-taking (*PropRisk*) in the *CombinedValence* loss trials correlated with risk-taking in *GainAlone* context, and risk-taking (*PropRisk*) in the *CombinedValence* gain trials correlated with risk-taking in *LossAlone* context (r > 0.6, p<0.003 in both cases]. Between subjects *ImpValence* in the *CombinedValence* context correlated with an analogous measure calculated by comparing *PropRisk* in *GainAlone* and *LossAlone* contexts [r = 0.56, p = 0.003].

#### Reaction times

We again replicated the RT findings in the *CombinedValence* context, both for valence (gains 485 ± 100 ms; losses, 560 ± 109 ms; *t_(25)_* = -6.3, p < 0.001); and for risk (*PropRisk_gain_* predicted the RT bias with gains, r = -0.6, p = 0.004; and *PropRisk_loss_* predicted the RT bias with losses, r = -0.7, p < 0.001). In the separated valence contexts, regarding valence RTs did not differ between contexts (*GainAlone* 462 ± 88 ms; *LossAlone*, 480 ± 112 ms; *t_(25)_* = -0.8, p = 0.4); and regarding risk the same correlation was seen with gains (*GainAlone*, r = -0.6, p = 0.003) and a trend with losses (*LossAlone*, r = -0.4, p = 0.062).

The proportion of missed trials was low (1 ±1%) and unaltered between tasks (CombinedValence 2 ±2%, GainAlone1 ±1%, LossAlone 1 ±1%, one way ANOVA, F_(20.4/7)_ = 2.9, p = 0.07).

#### Modelling of choice

We replicate our findings in the *CombinedValence* context (i.e. the standard accept/reject task). Variance and valence consistently influenced choice, shown by the mean-variance valence model winning that includes both (MO BIC=5577; MV 4694; MVV 4637). It correctly predicted 81%±8% of choices. In the contexts with only one trial type (i.e the MVV could not be used), as expected incorporating risk improved the model (*GainAlone* context [MO 5585, MV 3890]; *LossAlone* [MO 5291 MV 4369]) and this MV model well predicted actual choices (*GainAlone* 85%±7%, *LossAlone* 82%±9%).

## Experiment 3

### Results

#### Modelling of choice

We replicate the findings for EV, risk and valence for both tasks as shown above in Experiment 1, and also here when each block type in these experiments is analysed separately.

## References

Wright ND, Symmonds M, Hodgson K, Fitzgerald THB, Crawford B, Dolan RJ (2012) Approach–Avoidance Processes Contribute to Dissociable Impacts of Risk and Loss on Choice. J Neurosci 32:7009–7020.

## Supplementary figures

**
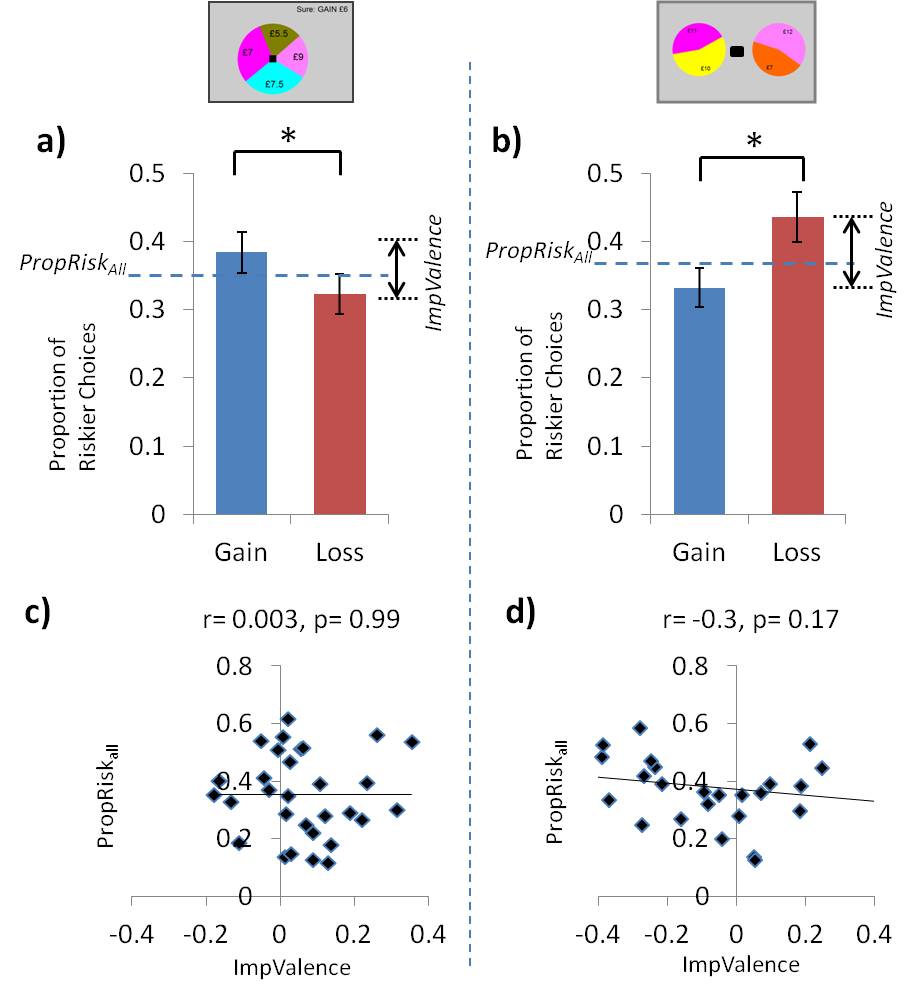
**

**Figure S1** **Experiment 1** Choice behaviour across subjects is shown in the accept/reject (panel a) and selection (panel b) tasks. In neither task did the influence of risk on an individual’s choices (PropRisk_all_) predict the impact of valence (ImpValence) (panels c and d respectively).


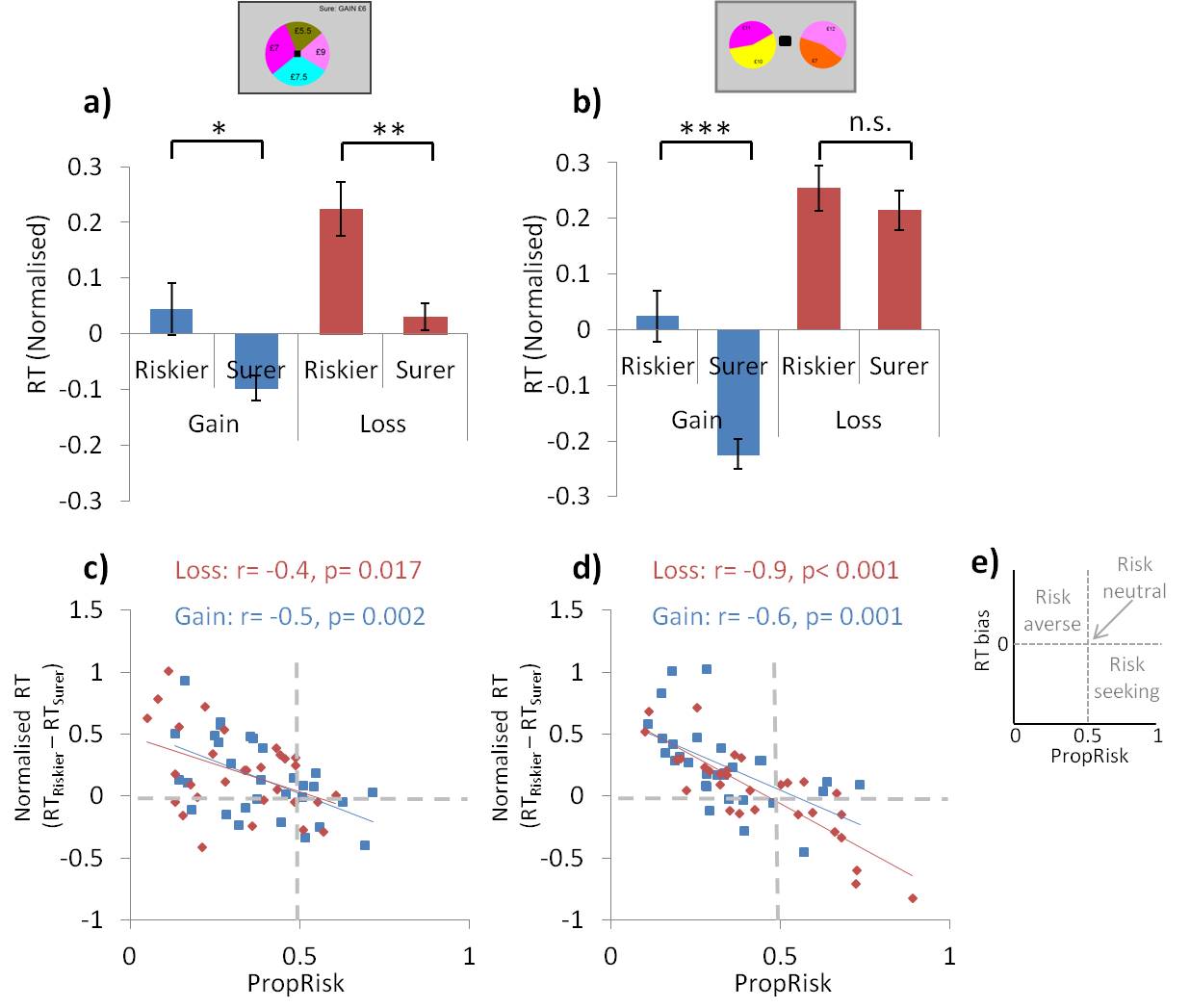


**Figure S2 Experiment 1 Reaction times** RTs replicate previous data (full descriptions in Wright et al., 2012) and are consistent with approach-avoidance processes in the accept/reject task (a,c) and selection task (c,d). e) is a cartoon of the RT predictions with risk preference. Error bars SEM. *p < 0.05; ** p < 0.005; *** p < 0.0005.

**Figure S3. Experiments 3a and 3b** **Dissociable influences of risk and valence on choice** Accept/reject task panels a and b; selection task panels c and d. Collapsing across the go-nogo manipulation we show the same dissociations as in Experiment 1, both across subjects (a, c) and between subjects (b, d).Error bars SEM. **p < 0.005.
